# Association of the Magnitude of Anti-SARS-CoV-2 Vaccine Side Effects with Sex, Allergy History, Chronic Diseases, Medication Intake, and SARS-CoV-2 Infection

**DOI:** 10.3390/vaccines12010104

**Published:** 2024-01-20

**Authors:** Elias A. Said, Afnan Al-Rubkhi, Sanjay Jaju, Crystal Y. Koh, Mohammed S. Al-Balushi, Khalid Al-Naamani, Siham Al-Sinani, Juma Z. Al-Busaidi, Ali A. Al-Jabri

**Affiliations:** 1 Department of Microbiology and Immunology, College of Medicine and Health Sciences, Sultan Qaboos University, P.O. Box 35, Muscat 123, Oman; s129895@student.squ.edu.om (A.A.-R.);; 2 Department of Family Medicine and Public Health, College of Medicine and Health Sciences, Sultan Qaboos University, P.O. Box 35, Muscat 123, Oman; sanjay@squ.edu.om; 3 Department of Medicine, Armed Forces Hospital, P.O. Box 726, Muscat 111, Oman; 4 Oman Medical Specialty Board, P.O. Box 1948, Muscat 130, Oman

**Keywords:** vaccine, SARS-CoV-2, BNT162b2, ChAdOx1-S, side effect

## Abstract

Vaccination provides the best protection against the increasing infections of SARS-CoV-2. The magnitude and type of anti-SARS-CoV-2 vaccine side effects (SEs) depend on parameters that are not fully understood. In this cross-sectional study, the associations between different anti-SARS-CoV-2 vaccine SEs and age, sex, the presence of chronic diseases, medication intake, history of allergies, and infections with SARS-CoV-2 were investigated. Our survey used the Google platform and had 866 participants, contacted through e-mails, social media and chain referral sampling (margin of error ≈ 4.38%, 99% confidence). More than 99% of the participants received the BNT162b2 and ChAdOx1-S vaccines. Being female, having chronic diseases, taking medicines routinely and the presence of a SARS-CoV-2 infection (*p* < 0.05) were associated with strong SEs after the BNT162b2 vaccine second dose. Having a history of allergies and a female sex (*p* < 0.01) were associated with strong SEs after the ChAdOx1-S vaccine second dose. Furthermore, the results reveal, for the first time, the associations between having a history of allergies, chronic diseases, medication usage, and SEs of a strong magnitude for the BNT162b2 and ChAdOx1-S vaccines. Additionally, this study supports the association of the female sex and infection with SARS-CoV-2 with an increased potential of developing stronger SEs with certain anti-SARS-CoV-2 vaccines.

## 1. Introduction

Coronavirus disease 2019 (COVID-19) is caused by infection with the severe acute respiratory syndrome coronavirus 2 (SARS-CoV-2), which mainly affects the respiratory system [1]. SARS-CoV2 belongs to the Coronaviridae family that includes HCoV-229E, HCoV-NL63, HCoV-HKU1, and HCoV-OC43 [1]. COVID-19 has caused a very high number of deaths; more than six and a half million people have died as of the date of this article’s preparation [2]. With the increase in the number of infections with SARS-CoV-2 variants [3], anti-SARS-CoV-2 vaccination provides the best protection against the virus [3].

The rapid spread of SARS-CoV-2, and the major impact that the infection has had on humans, has made the production of anti-SARS-CoV-2 vaccines an emergency [2]. Among the first and most commonly used anti-SARS-CoV-2 vaccines were the BNT162b2 (Pfizer-BioNTech, Mainz, Germany), mRNA-1273 (Moderna, Cambridge, MA, USA), ChAdOx1-S (Astra-Zeneca, Oxford, UK), Ad26.COV2. S (Johnson and Johnson, New Brunswick, NJ, USA), Sputnik V, Sinovac-Coronavac, and Sinopharm (Beijing, China) vaccines [4]. The BNT162b2 and mRNA-1273 vaccines are mRNA-based vaccines that are injected intramuscularly as lipid nanoparticles (LNPs) with a formulated mRNA. LNPs attach to host cells and release its mRNA to the cytoplasm, and then the mRNA is translated to the viral spike protein. These vaccines had an efficacy of around 95% for BNT162b2 and 94.5% for mRNA-1273 [4,5]. The ChAdOx1-S, Ad26.COV2. S, and Sputnik V vaccines use adenovirus vectors to deliver the SARS-CoV-2 spike protein to induce immune responses against it [4,6]. Sinovac-Coronavac and Sinopharm vaccines use inactivated SARS-CoV-2 [4]. The efficacy of these vaccines has been lower than the mRNA-based vaccines [4,7].

Similar to any other vaccine, anti-SARS-CoV-2 vaccines induce side effects that range from mild to strong. The most common side effects of the anti-SARS-CoV-2 vaccines have included injection site pain, headaches, flu-like symptoms, fever, and fatigue. Less common side effects have included fast heartbeat, whole body aches, difficulty breathing, joint pain, chills, stomachache, diarrhea, dizziness, and drowsiness. Rare side effects have included Bell’s palsy, thrombosis, myocarditis, allergy, numbness, lymph node swelling, and tenderness [8,9,10,11]. Moreover, a risk of death upon vaccination with the BNT162b2, mRNA-1273, and ChAdOx1-S vaccines has been reported [12,13]. The overall mortality was very low for the RNA vaccines [13]. Other studies have also reported a low risk of death following vaccination with the BNT162b2, mRNA-1273, and ChAdOx1-S vaccines [14,15]. The variation in the type and severity of the side effects depends on many parameters that are not completely known and understood. These parameters might include vaccine type, age, sex, number of doses, presence of diseases, medication intake, and previous infection with SARS-CoV-2 [16,17]. Other factors such as diabetes [18,19], body mass index (BMI) [20], and nutrients including vitamin D, Omega-3 fatty acids, and zinc [20,21] may also affect the side effects of anti-SARS-CoV-2 vaccines and the related immune responses [21,22,23]. Determining the parameters associated with vaccine side effects, which will allow a better understanding of the vaccine reactivity and mechanisms that govern the occurrence of these side effects, improvement of vaccine preparations, and increased community acceptance [16,17]. To our knowledge, previous studies have not investigated the presence of an association between the magnitude of the side effects of anti-SARS-CoV-2 vaccines and many of these potential parameters, especially having a history of allergy, the presence of chronic diseases, and taking medication. In this study, the presence of associations between the side effects of anti-SARS-CoV-2 vaccines and different parameters, including age, sex, the presence of chronic diseases, medication intake, the presence of a history of allergies, and infections with SARS-CoV-2, was assessed.

## 2. Methods

### 2.1. Study Design and Settings

A prospective cross-sectional study using an online survey was conducted between 30 June 2022 and 3 February 2023 among the residents of Oman. The study used a questionnaire to document the side effects reported upon the reception of different anti-SARS-CoV-2 vaccines and other parameters that may affect the side effects of these vaccines. No personal information that would permit the identification of the participant was collected. Each participant’s informed consent was obtained electronically and participants entered the survey only upon their agreement. All data are kept confidential. The study was approved by the Medical Research Ethics Committee (MREC#2784) in the College of Medicine and Health Sciences at the Sultan Qaboos University and the National Center for Statistics and Information in Oman (NCSI; MWAM 224215459).

### 2.2. The Questionnaire

The questionnaire was designed to record the side effects of the received anti-SARS-CoV-2 vaccines, including those mentioned above [8,9,10,11], and other parameters including sex, age, the presence of chronic diseases, medication intake, the presence of a history of allergies, and infection with SARS-CoV-2. The data were collected using Google Forms in two languages, either English (https://forms.gle/hwsujWXZcBDLMjXG6 (accessed on 2 October 2023)) (Supplementary Materials available on https://zenodo.org/records/10450867 (accessed on 2 January 2024)) or Arabic (https://forms.gle/jGSWsuHVa7sZ1wYe7) (Supplementary Materials available on https://zenodo.org/records/10450881 (accessed on 2 January 2024)). The questionnaire was pilot tested among 8 participants to assess its feasibility and reliability. The questionnaire was sent to participants through e-mails and social media. This included individuals in educational and health institutions, companies, and associations. The “chain referral sampling” or “snowball sampling” method, where participants referred their friends, colleagues, or acquaintances to the survey, was also used. These combined approaches were used to reach a wide range of potential participants, maximizing the likelihood of obtaining a diverse and representative sample that includes individuals from different backgrounds, experiences, and perspectives. The chain referral sampling approach allows reaching individuals who may be more difficult to identify or access through other methods.

### 2.3. Study Participants and Sample Size

The participants were 18 years old and above, of any nationality, residing in Oman, and had received one or more doses of different anti-SARS-CoV-2 vaccines in Oman or other countries. The number of individuals who accessed the survey was 866, among which 841 consented and participated.

Oman had a population of approximately 4.6 million people when this survey was conducted [24], and around 63.8% of the total population had received at least one dose of the anti-SARS-CoV-2 vaccines [25]. Therefore, with 866 participants, the margin of error is ≈4.38% for a 99% confidence and ≈3.33% for a 95% confidence.

### 2.4. Classification of the Side Effect Categories

The side effects were classified according to their magnitude in the following categories: absent (none), mild, and strong side effects. The side effects were considered strong if they were intense, affected or presented a threat to the life of the individual or the essential functions of their body parts, or might have significantly interfered with the normal activity of the individual. These included fever > 38 °C, flu-like symptoms, enlarged lymph nodes, allergy, thrombosis, myocarditis, fast heartbeat, erythema nodosum, difficulty in breathing, and Bell’s palsy; the other reported symptoms were considered mild.

### 2.5. Data Analysis

The chi-squared test was used to calculate the *p* value in order to assess the significance of the associations of the magnitude of the anti-SARS-CoV-2 vaccination side effects with the different parameters, including their association with SARS-CoV-2 infection. A *p* value < 0.05 was considered significant. The odds ratio (OR) was also calculated. The Statistical Package for the Social Sciences (SPSS) software version 23 was used in our data analysis.

## 3. Results

### 3.1. Characteristics of the Participants

Among the 866 registered responses, 841 (97.1%) accepted to participate in the study. Females represented 73.25% of the participants (616 individuals); the age of 431 participants (51.4%) was between 18 and 29 years (Supplementary Table S1). Among the participants, 136 (11.89%) declared having a chronic disease, and the most common disease among participants was hypertension (23 participants; Supplementary Table S1). The number and percentage of participants who had received one dose, two doses, three doses, or more than three doses of an anti-SARS-CoV-2 vaccine were 12 (1.43%), 674 (80.14%), 148 (18.60%), and 3 (0.36%), respectively (Supplementary Table S2). The majority of the participants declared receiving the BNT162b2 vaccine in the first (702 participants), second (673 participants), and third (101 participants) doses (Supplementary Table S2). This was followed by the ChAdOx1-S vaccine, which was administrated to 92 participants in the first dose, 98 in the second dose, and 11 in the third dose (Supplementary Table S2).

### 3.2. The Side Effects (SEs) of the Vaccines

**Figure 1:**
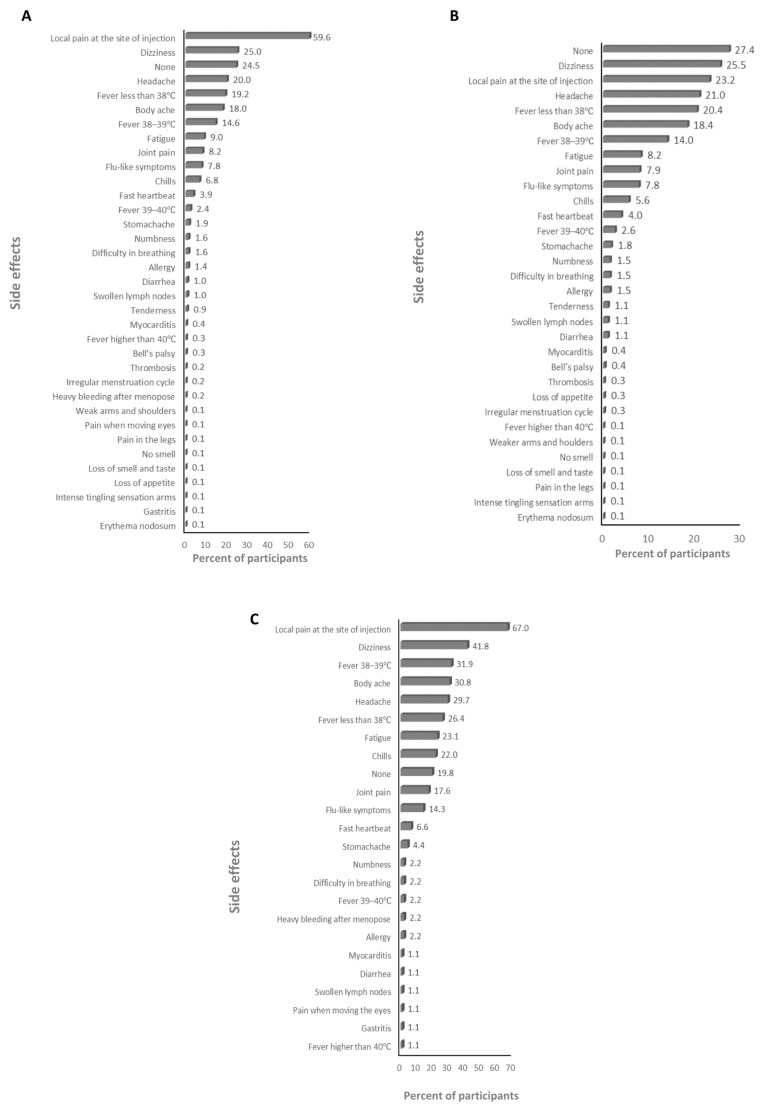
The frequency of side effects declared after the first dose: (**A**) for SARS-CoV-2 vaccines overall, (**B**) for the BNT162b2 vaccine, and (**C**) for the ChAdOx1-s vaccine.

**Figure 2:**
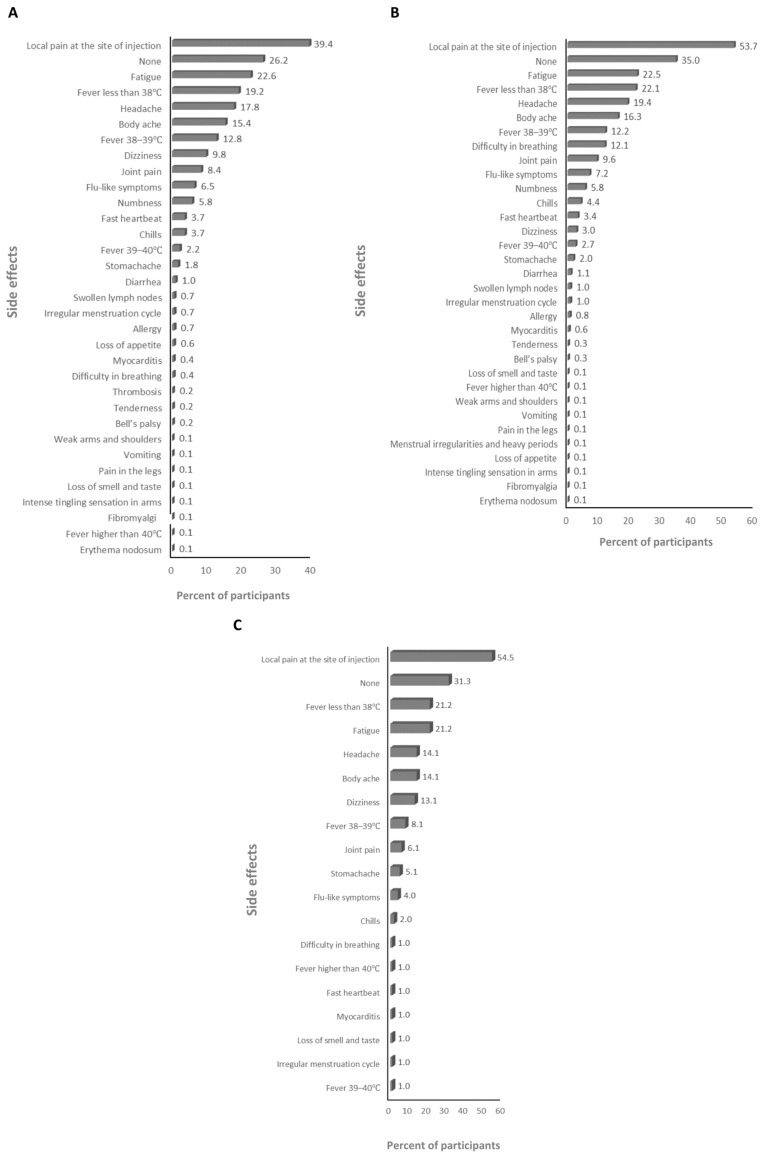
The frequency of side effects declared after the second dose: (**A**) for SARS-CoV-2 vaccines overall, (**B**) for the BNT162b2 vaccine, and (**C**) for the ChAdOx1-s vaccine.

**Figure 3:**
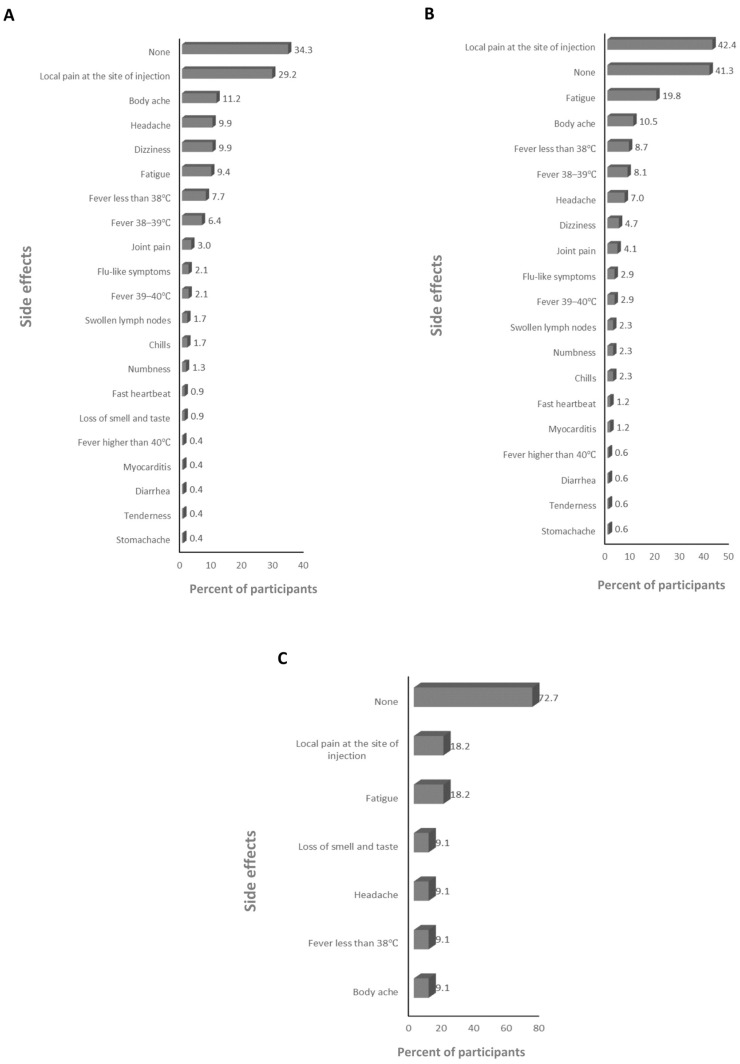
The frequency of side effects declared after the third dose: (**A**) for SARS-CoV-2 vaccines overall, (**B**) for the BNT162b2 vaccine, and (**C**) for the ChAdOx1-s vaccine.

The absolute majority of participants received the BNT162b2 and ChAdOx1-S vaccines (>99%; Supplementary Table S2), therefore the frequency of the side effects for all of the anti-SARS-CoV-2 vaccines and for just the BNT162b2 and ChAdOx1-S vaccines was assessed. The main side effects that participants declared after the first dose were local pain at the site of injection and dizziness for the anti-SARS-CoV-2 vaccines overall (59.6% and 25%, respectively; Figure 1A), and the BNT162b2 (23.2% and 25.5%, respectively; Figure 1B), and ChAdOx1-S (67% and 41.8%, respectively; Figure 1C) vaccines. The main side effects that participants declared after the second dose were local pain at the site of injection and fatigue for the anti-SARS-CoV-2 vaccines overall (39.4% and 22.6%, respectively; Figure 2A) and the BNT162b2 vaccine (53.7% and 22.5%, respectively; Figure 2B), and local pain at the site of injection and fever less than 38 °C for the ChAdOx1-S vaccine (54.5% and 21.2%, respectively; Figure 2C). The main side effects that participants declared after the third dose were local pain at the site of injection and body ache for the anti-SARS-CoV-2 vaccines overall (29.2% and 11.2%, respectively; Figure 3A), and local pain at the site of injection and fatigue for the BNT162b2 (42.4% and 19.8%, respectively; Figure 3B) and ChAdOx1-S (72.7.5% and 18.2%, respectively; Figure 3C) vaccines.

The associations between the magnitude of the side effects of different anti-SARS-CoV-2 vaccines and sex, age, having a history of allergy, the presence of chronic diseases, autoimmune diseases, and routinely taking medications were assessed.

### 3.3. Parameters Associated with the Magnitude of the Side Effects of the Anti-SARS-CoV-2 Vaccines

**Figure 4:**
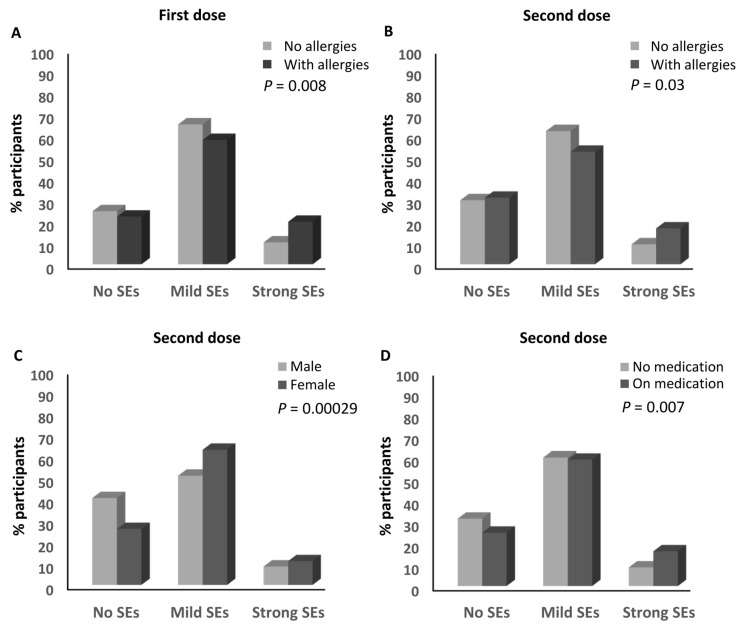
Factors associated with the magnitude of side effects of anti-SARS-CoV-2 vaccines. (**A**) Association between the magnitude of side effects after the first dose of an anti-SARS-CoV-2 vaccine and the presence of a history of allergy. (**B**) Association between the magnitude of side effects after the second dose of an anti-SARS-CoV-2 vaccine and the presence of a history of allergy. (**C**) Association between the magnitude of side effects after the second dose of an anti-SARS-CoV-2 vaccine and the sex of the participants. (**D**) Association between the magnitude of side effects after the second dose of an anti-SARS-CoV-2 vaccine and medication intake. SEs: side effects.

A higher percentage of the participants who declared having history of allergies (19.8%) developed strong side effects after the first dose of an anti-SARS-CoV-2 vaccine compared to those with no history of allergies (10.2%; ≈1.94-fold; *p* = 0.008; Table 1 and Figure 4A). No other parameters were associated with the magnitude of the side effects from the first dose (Table 1).

A similar association was also observed after the second dose of the anti-SARS-CoV-2 vaccines, where 16.7% of the participants who had a history of allergies developed strong side effects compared to 9.3% (≈1.8-fold) of those who did not have any history of allergies (*p* = 0.03; Table 1 and Figure 4B). Moreover, the sex of the participants was associated with the magnitude of the side effects they experienced after the second dose, where the percentage of females who developed strong side effects (11.1%) was higher than that of the males (8.6%; ≈1.3-fold; *p* = 0.000299; Table 1 and Figure 4C). In addition, a higher percentage of the participants who declared taking medications routinely (16.2%) developed strong side effects after the second dose compared to those who were not taking any routine medications (8.7%; ≈1.9-fold; *p* = 0.007; Table 1 and Figure 4D). No other parameters were associated with the magnitude of the side effects in regards to the second dose, and no parameters were associated with the magnitude of the side effects after the third dose (Table 1).

The absolute majority of participants received the BNT162b2 and ChAdOx1-S vaccines (>99%; Supplementary Table S2); therefore, the presence of associations between the magnitude of side effects after receiving these types of vaccines and the different parameters was investigated.

### 3.4. Parameters Associated with the Magnitude of Side Effects for the BNT162b2 Vaccine

No parameter was significantly associated with the magnitude of the side effects after the first dose of the BNT162b2 vaccine (Table 2). Although, there was a tendency of a higher percentage of participants who reported having a history of allergies (18.3%) to develop strong side effects after the first dose, compared to 10.4% (≈1.8-fold) of those without any history of allergies (*p* = 0.07; Table 2).

**Figure 5:**
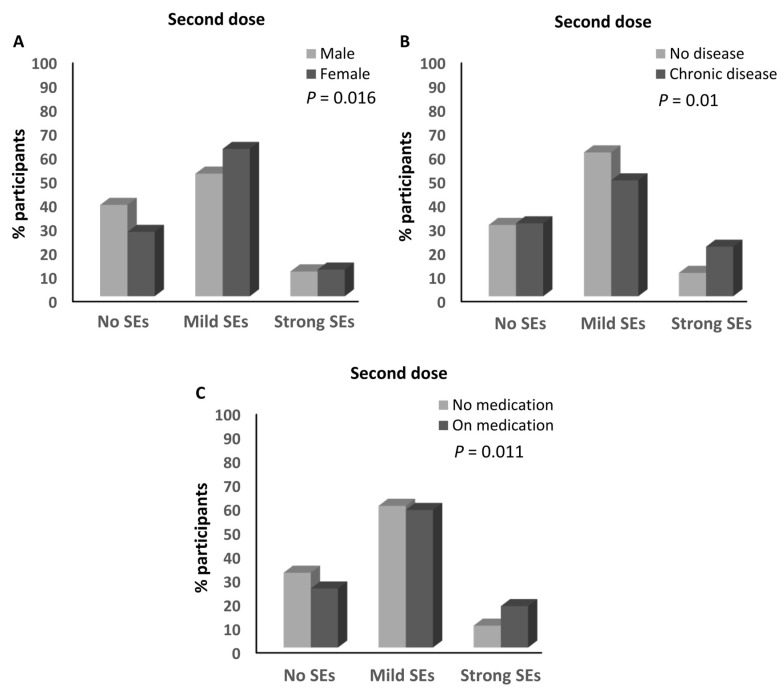
Factors associated with the magnitude of side effects of the BNT162b2 vaccine. (**A**) Association between the magnitude of the side effects after the second dose of the BNT162b2 vaccine and the sex of the participants. (**B**) Association between the magnitude of the side effects after the second dose of the BNT162b2 vaccine and the presence of chronic diseases. (**C**) Association between the magnitude of the side effects after the second dose of the BNT162b2 vaccine and medication intake. SEs: side effects.

However, sex, having chronic diseases, and taking regular medications were significantly associated with the magnitude of the side effects after receiving the second dose of the BNT162b2 vaccine. In fact, a higher percentage of females developed mild (61.8%) and strong (11.2) side effects compared to the males with mild (51.4%; ≈1.2-fold) and strong (10.4%; ≈1.1-fold) side effects (*p* = 0.016; Table 2 and Figure 5A). Moreover, a higher percentage of participants who reported having chronic diseases (20.8%) developed strong side effects compared to those who did not have chronic diseases (9.8%; ≈2.12-fold; *p* = 0.01; Table 2 and Figure 5B). Furthermore, a higher percentage of participants who took medicines routinely (17.4%) developed strong side effects compared to those not taking medications (9.2%; ≈1.9-fold; *p* = 0.01; Table 2 and Figure 5C). No other parameters were associated with the magnitude of side effects after the second dose of the BNT162b2 vaccine (Table 2). Moreover, no parameters were associated with the magnitude of side effects after the third dose of the BNT162b2 vaccine (Table 2).

### 3.5. Parameters Associated with the Magnitude of Side Effects of the ChAdOx1-S Vaccine

**Figure 6:**
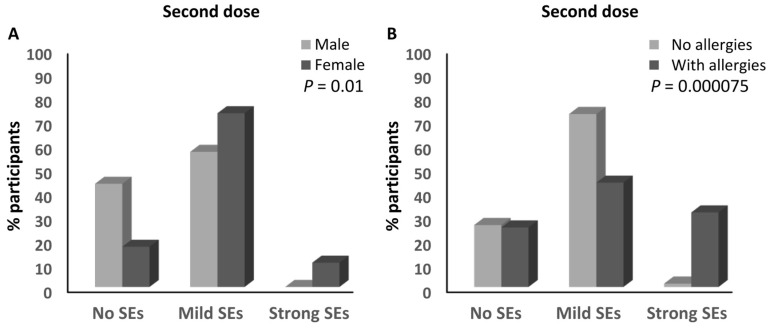
Factors associated with the magnitude of side effects of the ChAdOx1-S vaccine. (**A**) Association between the magnitude of the side effects after the second dose of the ChAdOx1-S vaccine and the sex of the participants. (**B**) Association between the magnitude of the side effects after the second dose of the ChAdOx1-S vaccine and the presence of a history of allergies. SEs: side effects.

No parameters were significantly associated with the magnitude of the side effects after the first dose of the ChAdOx1-S vaccine (Table 3). However, sex and having a history of allergies were significantly associated with the magnitude of the side effects after receiving the second dose of the ChAdOx1-S vaccine. Similar to the BNT162b2 vaccine, a higher percentage of females developed mild (72.9%) and strong (10.2%) side effects compared to the males with mild (56.7%; ≈1.3-fold) and strong (0%) side effects after the second dose of the ChAdOx1-S vaccine (*p* = 0.01; Table 3 and Figure 6A). Moreover, 31.3% of the participants with a history of allergies developed strong side effects compared to 1.4% (≈22.4-fold) of those without any history of allergies (*p* = 0.000075; Table 3 and Figure 6B).

The number of participants that received a third dose of the ChAdOx1-S vaccine was very small, therefore the presence of associations with the magnitude of its side effects was not investigated.

### 3.6. Association between Having a History of Allergies and Developing Allergies upon Vaccination

It has been reported that individuals with a history of allergies before vaccination were significantly more likely to experience allergic reactions after vaccination [26]. Therefore, the presence of associations between having a history of allergies and developing an allergic reaction upon vaccination was investigated. A higher percentage of participants who reported having a history of allergies (4.76%) developed allergic reactions to the first dose of the anti-SARS-CoV-2 vaccines compared to participants who did not have a history of allergy (0.56%; ≈8.5-fold; *p* = 0.000062; Supplementary Table S3). This was also documented after the second doses of different anti-SARS-CoV-2 vaccines. In fact, a higher percentage of participants who reported having a history of allergies (2.38%) developed allergic reactions to the second dose compared to participants who did not have a history of allergies (0.57%; ≈4.2-fold; *p* = 0.041; Supplementary Table S3). In addition, and contrary to the fact that having a history of allergies was not associated with the magnitude of the side effects after the BNT162b2 vaccine (reported above), a higher percentage of participants who had a history of allergies (4.81%) developed allergic reactions to the first dose of the BNT162b2 vaccine compared to participants who did not have a history of allergies (0.5%; ≈9.62-fold; *p* = 0.000134; Supplementary Table S3). Moreover, higher percentage of participants who had a history of allergy (2.88%) developed allergic reactions to the second dose of the BNT162b2 vaccine compared to participants who did not have a history of allergy (0.51%; ≈5.65-fold; *p* = 0.016; Supplementary Table S3). In contrast, although having a history of allergies was associated with the magnitude of side effects after the second dose of the ChAdOx1-S vaccine (reported above), no associations were detected between having a history of allergies and developing allergic reactions upon the vaccination with the first two doses of the ChAdOx1-S vaccine (Supplementary Table S3).

No participants declared developing an allergic reaction upon the third dose of any of the anti-SARS-CoV-2 vaccines.

### 3.7. Infection with SARS-CoV-2 and the Magnitude of Side Effects after the Anti-SARS-CoV-2 Vaccination

Among the participants, 314 declared that they were infected with SARS-CoV-2 once, 113 declared that they were infected twice, and 401 participants did not report any infection with the virus. The presence of associations between SARS-CoV-2 infection and the magnitude of side effects after receiving an anti-SARS-CoV-2 vaccination was investigated.

**Figure 7:**
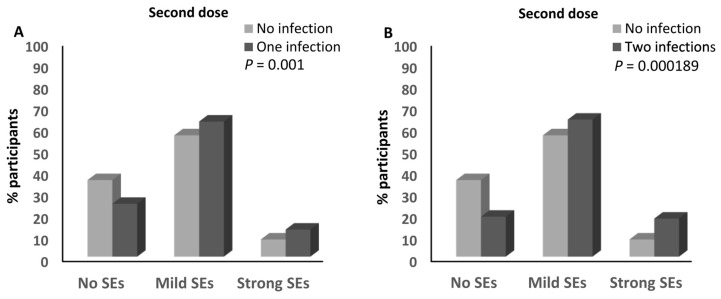

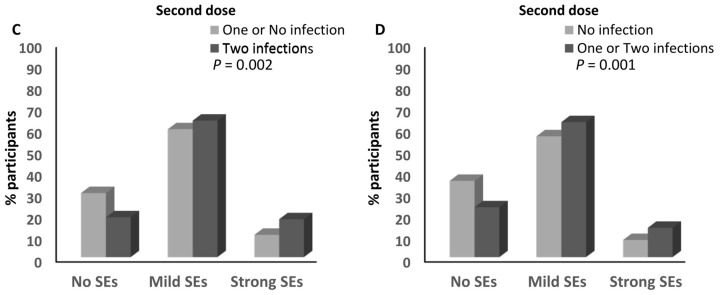
Association between SARS-CoV-2 infection and the magnitude of side effects after the anti-SARS-CoV-2 vaccination. (**A**) Comparison of the magnitude of side effects after the second doses of the anti-SARS-CoV-2 vaccines between participants who declared no infection with SARS-CoV-2 and those who declared one infection with the virus. (**B**) Comparison of the magnitude of side effects after the second doses of the anti-SARS-CoV-2 vaccines between participants who declared no infection with SARS-CoV-2 and those who declared two infections with the virus. (**C**) Comparison of the magnitude of side effects after the second doses of the anti-SARS-CoV-2 vaccines between participants who declared one or no infection with SARS-CoV-2 and those who declared two infections with the virus. (**D**) Comparison of the magnitude of side effects after the second doses of the anti-SARS-CoV-2 vaccines between participants who declared no infection with SARS-CoV-2 and those who declared one or two infections with the virus. SEs: side effects.

No association was detected between the number of infections and the magnitude of the side effects after the first dose of any of the different anti-SARS-CoV-2 vaccines (Supplementary Table S4). In contrast, the percentage of participants who reported having one infection with SARS-CoV-2 and had mild (62.8%) and strong (12.6%) side effects after the second dose of the anti-SARS-CoV-2 vaccine was higher than that of the participants who had mild (56.4%; ≈1.1-fold) and strong (8%; ≈1.6-fold) side effects after the second dose of the SARS-CoV-2 vaccine and who did not report any previous infection with the virus (*p* = 0.001; Figure 7A and Supplementary Table S4). Moreover, the percentage of participants who reported two previous infections with SARS-CoV-2 and had mild (63.7%) and strong (17.7%) side effects after the second dose of the anti-SARS-CoV-2 vaccine was higher than that of the participants who had mild (56.4%; ≈1.13-fold) and strong (8%; ≈2.2-fold) side effects and did not report any previous infection with SARS-CoV-2 (*p* = 0.000189; Figure 7B and Supplementary Table S4). It was also higher than that of both participants who had mild (59.7%; ≈1.1-fold) and strong (10.4%; ≈1.7-fold) side effects and reported one or no previous infection with the virus (*p* = 0.002; Figure 7C and Supplementary Table S4). In addition, the percentage of participants who reported one or two infections with SARS-CoV-2 and had mild (63%) and strong (13.7%) side effects after the second dose of the anti-SARS-CoV-2 vaccine was higher than that of the participants who had mild (56.4%; ≈1.12-fold) and strong (8%; ≈1.7-fold) side effects and did not report any previous infection with SARS-CoV-2 (*p* = 0.001; Figure 7D and Supplementary Table S4). There was a trend that showed higher percentages of participants who had mild (63.7%) and strong (17.7%) side effects after the second dose of the anti-SARS-CoV-2 vaccine among those who reported having two previous infections with the virus compared to those who reported one infection and had mild (62.9%) and strong (12.6%) side effects after the second dose of the vaccine (*p* = 0.068; Supplementary Table S4). Moreover, the percentage of participants who had experienced two infections with SARS-CoV-2 and developed strong side effects after the third dose of the anti-SARS-CoV-2 vaccine (14.8%) was higher than that of those who reported no experience of infection with the virus (1.6%; ≈9.25-fold; *p* = 0.039; Supplementary Table S4). No other associations were detected between the number of SARS-CoV-2 infections and the magnitude of the side effects after the third dose of the anti-SARS-CoV-2 vaccine (Supplementary Table S4).

### 3.8. Infection with SARS-CoV-2 and the Magnitude of Side Effects after the BNT162b2 Vaccine

No association was detected between the number of SARS-CoV-2 infections and the magnitude of side effects after the first dose of the BNT162b2 vaccine (Supplementary Table S5).

**Figure 8:**
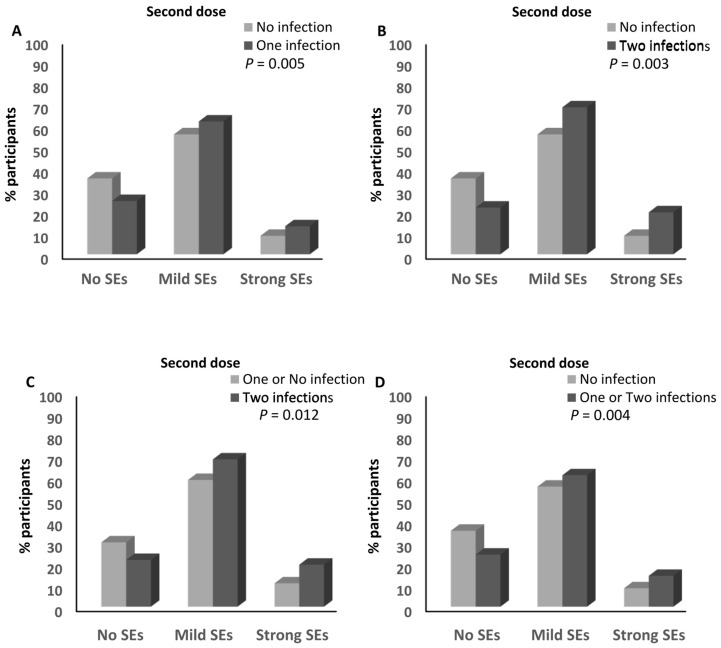
Association between SARS-CoV-2 infection and the magnitude of side effects after the BNT162b2 vaccine. (**A**) Comparison of the magnitude of side effects after the second dose of the BNT162b2 vaccine between participants who declared no infection with SARS-CoV-2 and those who declared one infection with the virus. (**B**) Comparison of the magnitude of side effects after the second dose of the BNT162b2 vaccine between participants who declared no infection with SARS-CoV-2 and those who declared two infections with the virus. (**C**) Comparison of the magnitude of side effects after the second dose of the BNT162b2 vaccine between participants who declared one or no infection with SARS-CoV-2 and those who declared two infections with the virus. (**D**) Comparison of the magnitude of side effects after the second dose of the BNT162b2 vaccine between participants who declared no infection with SARS-CoV-2 and those who declared one or two infections with the virus. SEs: side effects.

However, the percentage of participants who reported one infection with SARS-CoV-2 and had mild (62%) and strong (13.1%) side effects after the second dose of the BNT162b2 vaccine was higher than that of the participants who had mild (56%; ≈1.1-fold) and strong (8.6%; ≈1.5-fold) side effects and did not report any previous infection with SARS-CoV-2 (*p* = 0.005; Figure 8A and Supplementary Table S5). Moreover, the percentage of participants who reported two infections with SARS-CoV-2 and had mild (68.6%) and strong (19.6%) side effects after the second dose of the BNT162b2 vaccine was higher than the percentage of participants who had mild (56%; ≈1.2-fold) and strong (8.6%; ≈2.3-fold) side effects and did not report any previous infection with SARS-CoV-2 (*p* = 0.003; Figure 8B and Supplementary Table S5), and the percentages of both participants who had mild (59.1%; ≈1.1-fold) and strong (10.9%; ≈1.8-fold) side effects and reported one or no infection with the virus (*p* = 0.012; Figure 8C and Supplementary Table S5). In addition, the percentage of participants who reported one or two infections with SARS-CoV-2 and had mild (61.3%) and strong (14.4%) side effects after the second dose of the BNT162b2 vaccine was higher than that of the participants who had mild (56%; ≈1.1-fold) and strong (8.6%; ≈1.7-fold) side effects and did not report any previous infection with SARS-CoV-2 (*p* = 0.004; Figure 8D and Supplementary Table S5). There was no association between the magnitude of side effects and reporting two infections with the virus (Supplementary Table S5). The number of infections with SARS-CoV-2 was not associated with the magnitude of the side effects of the third dose of the BNT162b2 vaccine (Supplementary Table S5).

### 3.9. Infection with SARS-CoV-2 and the Magnitude of Side Effects after the ChAdOx1-S Vaccine

No association was detected between the number of infections and the magnitude of the side effects after the first and second doses of the ChAdOx1-S vaccine (Supplementary Table S6). The number of participants receiving the third dose of the ChAdOx1-S vaccine was too small to investigate the presence of any associations.

## 4. Discussion

This study reports on the presence of significant associations between several parameters and the magnitude of side effects of anti-SARS-CoV-2 vaccines, especially the BNT162b2 and ChAdOx1-S vaccines. Having a history of allergies was associated with the magnitude of side effects after the first dose of the anti-SARS-CoV-2 vaccines, while having a history of allergies, being female, taking regular medications, and being infection with SARS-CoV-2 were associated with experiencing magnitude of side effects after the second dose of the anti-SARS-CoV-2 vaccine. Although no parameters were associated with the magnitude of the side effects after the first dose of the BNT162b2 and ChAdOx1-S vaccines, being female, having chronic diseases, taking medicines routinely, and having a history of SARS-CoV-2 infection were associated with the magnitude of side effects after the second dose of the BNT162b2 vaccine, and having a history of allergy and being female were associated with the magnitude of side effects after the second dose of the ChAdOx1-S vaccine. Previous studies have reported the presence of associations between: 1. having a history of allergies and having an allergic reaction to the vaccine [26], local side effects [27], and non-allergic side effects [26]; 2. female sex and a higher magnitude of side effects [16,28,29]; 3. the presence of chronic diseases and the development of side effects as discussed below; and 4. previous infections with SARS-CoV-2 and the development of strong side effects [30,31] after anti-SARS-CoV-2 vaccination. Further details about these comparisons are provided in the following paragraphs.

Our results indicate that female sex is associated with a higher magnitude of side effects after the second dose of the anti-SARS-CoV-2 vaccines for all reported vaccines when their results were analyzed together and for the BNT162b2 and ChAdOx1-S vaccines when their results were analyzed individually. These results support previous studies reporting an association between female sex and developing a higher magnitude of side effects after the BNT162b2 and ChAdOx1-S vaccines [16,28,29]. Such association was also reported with the side effects of anti-influenza virus vaccines, e.g., A/Cal/09 H1N1, the inactivated H1N1 vaccines (IV), and the live attenuated influenza vaccine (LAIV) [32,33]. This difference between males and females might be due to sex-associated biological variations, including genetics, hormones, immunological responses, and pharmacological aspects. Vaccines are designed to induce immune responses, and female and male immune systems have many differences [34,35]. In humans, many of the approximately 2000 genes in the X chromosome play crucial roles in regulating the immune system [34,35]. These genes include those of transcription factors, cytokine receptors and pattern recognition receptors (PRRs), and numerous microRNAs that contribute to the modulation of immune responses [34,35]. Although one of the X chromosomes is inactivated to assure the expression of only one set of X chromosome genes in females, around 15% of genes in humans can escape X inactivation. Therefore, females exhibit higher gene expression levels compared to males. This can result in distinct immune responses between males and females [34,35]. For instance, in females, the biallelic Toll-like receptor-7 (TLR-7) expression, which detects single-stranded RNA (ssRNA) [36], might mediate a stronger sensing of the vaccines and their product, especially the mRNA vaccine BNT162b2; this results in the induction of a stronger immune activation. Moreover, the male sex hormone dehydroepiandrosterone enhances the production of the immunoregulatory cytokine IL-10 upon triggering TLR-9, which detects CpG DNA [37]; this might result in a milder stimulation of the immune response to vaccines in males. However, the precise mechanisms that are directly responsible for the differences in the magnitude of side effects between males and females must be investigated.

The results of this study pointed also to the presence of associations between having a history of allergies and the magnitude of side effects after anti-SARS-CoV-2 vaccination, particularly the ChAdOx1-S vaccine. In fact, a history of allergy associated with stronger side effects after the ChAdOx1-S vaccine. Interestingly, having a history of allergies was not associated with the development of an allergic reaction upon receiving this vaccine. In contrast, although having a history of allergy was associated with the development of an allergic reaction upon receiving the BNT162b2 vaccine, having a history of allergy was not associated with the magnitude of side effects of this vaccine. This indicates that the association between the presence of a history of allergies and the magnitude of side effects is not dependent on its association with the development of an allergic reaction upon vaccination. It also suggests that such association is vaccine-dependent, which can result from the differences in the composition and mechanisms of the induced immune responses between the two vaccines [38]. Although previous studies have reported that the presence of a history of allergies was associated with the development of allergic reactions [26], the risk of developing local side effects [27], and the risk of developing non-allergic side effects [26] after anti-SARS-CoV-2 vaccination, to our knowledge, no other study has reported the presence of associations between having a history of allergies and developing strong side effects after these anti-SARS-CoV-2 vaccines. The precise reason for this association is not known, however it might result from a higher susceptibility of the immune system to components in the vaccine’s ingredients that can induce strong responses that do not involve the IgEs required for allergy [26]. It is important to highlight that only a small number of individuals develop allergic reactions to anti-SARS-CoV-2 vaccination [38].

Our results also revealed the association between the presence of one or two previous infections with SARS-CoV-2 and the development of strong side effects after vaccination with the second dose of the BNT162b2 vaccine. This is supported by similar results that have been reported by previous studies [30,31]. This might be due to the presence of a prior immune response to the SARS-CoV-2 proteins, which induces a stronger response upon vaccination. Moreover, it is possible that participants who did not report SARS-CoV-2 infections had non-symptomatic infections with the virus, therefore the fact that they did not report side effects after the vaccine might result from the mild immune responses they develop to the SASRS-CoV-2 components.

In this study, the presence of chronic diseases was associated with a stronger magnitude of side effects after the second dose of BNT162b2 vaccine. This supports the results of previous studies that reported associations between the presence of chronic diseases and the development of side effects after anti-SARS-CoV-2 vaccination [28,39]. However, to our knowledge, no other study has reported the presence of associations between having chronic diseases and the magnitude of side effects after anti-SARS-CoV-2 vaccination. It is possible to hypothesize that individuals with chronic diseases are more vulnerable to the responses induced upon vaccination, however further studies are required to identify the reasons of such an association. This might also provide rational for the association between routinely taking medication and the development of strong side effects after the second dose of the BNT162b2 vaccine. To our knowledge, no other study has reported a similar association. Our questionnaire inquired about participants’ health conditions, specifically whether they had type 1 or type 2 diabetes. Additionally, participants were given the opportunity to report any other existing diseases. Responses included conditions such as asthma, Hashimoto’s thyroiditis, glucose-6-phosphate dehydrogenase (G6PD) deficiency, sickle cell disease, psoriasis, polycystic ovary syndrome (PCOS), hypercholesterolemia, obesity, hepatitis B infection, migraines, bipolar disorder, gout, spondylarthritis, gastroesophageal reflux disease (GERD), and irritable bowel syndrome (IBS). Some of these diseases can independently influence immune responses and vaccine side effects, which might also be impacted by deficiencies in vitamin D or zinc [18,19,20,21,22,23]. However, the low frequency of participants in each category (≈0.2% for most of them) and the focus of this paper on the analysis of other parameters that could influence the side effects made it very challenging to assess the impact of each of these diseases on the magnitude of side effects. Further studies need to be conducted to investigate this crucial aspect.

An association between the magnitude of the side effects of anti-SARS-CoV-2 vaccination, especially fever, and the anti-SARS-CoV-2 antibodies, especially upon the first vaccine inoculation, has been described in the literature [40,41,42]. However, the lack of side effects did not mean an absence of a protective antibody response [43]. The settings of our study did not allow for collecting data about the participants’ antibody titers to investigate such an association.

Different studies have reported the low risk of mortality upon receiving anti-SARS-CoV-2 vaccines [12,13,14,15]; however, because of the nature of our survey-based study, it was impossible to collect information about death events that could have occurred upon anti-SARS-CoV-2 vaccination. Death due to SARS-CoV-2 infection has also been reported in a small fraction of fully vaccinated individuals. These patients had risk factors such as comorbidities and older age [44]. This was further confirmed by a study involving complete autopsy and microscopic analysis of patient organs [45]. Such results were also reported upon the occurrence of infections with different variants, including the Omicron variant, upon vaccination with the ChAdOx1, CoronaVac, and BNT162b2 vaccines [46,47].

## 5. Conclusions

Altogether, this study points to different vaccine type-dependent factors that are associated with the development of strong side effects after anti-SARS-CoV-2 vaccination. Its results highlight for the first time the association between having a history of allergies, chronic diseases, and taking medication and the development of strong side effects with some anti-SARS-CoV-2 vaccines. Our results also support the association of the female sex and SARS-CoV-2 infection with the development of stronger side effects after some anti-SARS-CoV-2 vaccines. The mechanisms that mediate these associations must be investigated and these factors must be taken into consideration when developing and improving vaccines and for anticipating the management of the side effects that occur after vaccination. Reducing vaccine side effects is also important for decreasing the hesitance of the population to get vaccinations. It is also important that future studies include clinical verification of patient reports, use paper-based questionnaires to include participants who do not have routine access to technology, include patients of all age categories, and include patient relatives and medical record reviews to account for the death events that could have occurred upon anti-SARS-CoV-2 vaccination.

## 6. Study Strengths

This study included 866 participants, which is ≈2.245-fold higher than the number (385) required to obtain a 95% confidence level and ≈1.31-fold higher than the number (664) required to obtain a 99% confidence level and 5% margin of error. Therefore, the margin of error is ≈4.38% for a 99% confidence level and ≈3.33% for a 95% confidence level.

## 7. Study Limitations

The results of the questionnaire were analyzed from the participants’ own reports with no clinical verification, which may influence their accuracy. The use of the Google platform may have skewed the results towards younger age demographics and those with routine access to technology. Although the combined methods that were used in recruiting the participants have many advantages, as mentioned in the Methods section, part of these methods might lead to sampling and selection bias. The study method did not allow the collection of information about the death events that could have occurred upon anti-SARS-CoV-2 vaccination. The survey was conducted online with no face-to-face interview. Only 5.47% of the participants were above 50 years old, which might affect the collected information.

## Figures and Tables

**Table 1:** Parameters’ association with the side effects of the anti-SARS-CoV-2 vaccines.

Parameters		Side Effect Magnitude; n (%)			Odds Ratio			*p* Value
		None	Mild	Strong	None/ Mild	None/ Strong	Mild/ Strong	
First dose								
Sex	Male	53 (23.7%)	148 (66.1%)	23 (10.3%)	1.08	0.87	0.81	0.680
	Female	151 (24.6%)	389 (63.3%)	75 (12.2%)				
Allergies	No	176 (24.7%)	464 (65.1%)	73 (10.2%)	1.01	0.46	0.46	0.008
	Yes	28 (22.2%)	73 (57.9%)	25 (19.8%)				
Chronic disease	No	182 (24.6%)	471 (63.7%)	86 (11.6%)	0.86	0.87	1.00	0.850
	Yes	22 (22%)	66 (66%)	12 (12%)				
Medications	No	159 (24.4%)	418 (64.1%)	75 (11.5%)	0.99	0.92	0.93	0.960
	Yes	45 (24.1%)	119 (63.6%)	23 (12.3%)				
Autoimmune disease	No	200 (24.2%)	530 (64.1%)	97 (11.7%)	1.51	1.94	1.28	0.750
	Yes	4 (33.3%)	7 (58.3%)	1 (8.3%)				
Age	18–50	193 (24.1%)	513 (64.1%)	94 (11.8%)	1.22	1.34	1.10	0.830
	>50	11 (28.2%)	24 (61.5%)	4 (10.3%)				
Second dose								
Sex	Male	90 (40.5%)	113 (50.9%)	19 (8.6%)	0.52	0.50	0.96	0.000299
	Female	158 (26.1%)	381 (62.9%)	67 (11.1%)				
Allergies	No	209 (29.8%)	428 (61%)	65 (9.3%)	1.21	0.58	0.48	0.03
	Yes	39 (31%)	66 (52.4%)	21 (16.7%)				
Chronic disease	No	219 (30%)	442 (60.5%)	69 (9.5%	1.13	0.54	0.48	0.05
	Yes	29 (29.6%)	52 (53.1%)	17 (17.3%)				
Medications	No	202 (31.4%)	385 (59.9%)	56 (8.7%)	0.80	0.43	0.53	0.01
	Yes	46 (24.9)	109 (58.9%)	30 (16.2)				
Autoimmune disease	No	247 (30.3%)	486 (59.6%)	83 (10.2%)	0.25	0.11	0.46	0.11
	Yes	1 (8.3%)	8 (66.7%)	3 (25%)				
Age	18–50	234 (29.7%)	475 (60.2%)	80 (10.1%)	1.50	0.80	0.53	0.32
	>50	14 (35.9%)	19 (48.9%)	6 (15.4%)				
Third dose								
Sex	Male	18 (40%)	24 (53.3%)	3 (6.7%)	0.53	0.93	1.78	0.224
	Female	28 (26.9%)	71 (68.3%)	5 (4.8%)				
Allergies	No	42 (33.9%)	74 (59.7%)	8 (6.5%)	0.34	-	-	0.06
	Yes	4 (16%)	21 (84%)	0 (0%)				
Chronic disease	No	38 (32.8%)	74 (63.8%)	4 (3.4%)	0.74	0.21	0.28	0.16
	Yes	8 (24.2%)	21 (63.6%)	4 (12.1%)				
Medications	No	31 (34.1%)	58 (63.73%)	2 (2.2%)	0.76	0.16	0.21	0.79
	Yes	15 (25.9%)	37 (63.8%)	6 (10.3%)				
Autoimmune disease	No	45 (31.25%)	91 (63.2%)	8 (5.6%)	0.51	-	-	0.62
	Yes	1 (20%)	4 (80%)	0 (0%)				
Age	18–50	40 (31.5%)	81 (63.8%)	6 (4.7%)	0.87	0.45	0.52	0.71
	>50	6 (27.3%)	14 (63.6%)	2 (9.1%)				

**Table 2:** Parameters’ association with the side effects of the BNT162b2 vaccine.

Parameters		Side Effect Magnitude; n (%)			Odds Ratio			*p* Value
		None	Mild	Strong	None/ Mild	None/ Strong	Mild/ Strong	
First dose								
Sex	Male	40 (21.6%)	125 (67.6%)	20 (10.8%)	1.30	1.10	0.84	0.42
	Female	134 (25.9%)	322 (62.3%)	61 (11.8%)				
Allergies	No	151 (25.3%)	385 (64.4%)	62 (10.4%)	0.95	0.50	0.53	0.07
	Yes	23 (22.1%)	62 (59.6%)	19 (18.3%)				
Chronic disease	No	156 (24.8%)	399 (63.5%)	73 (11.6%)	0.96	1.05	1.10	0.97
	Yes	18 (24.3%)	48 (64.9%)	8 (10.8%)				
Medications	No	135 (24.5%)	351 (63.7%)	65 (11.8%)	1.06	1.17	1.11	0.89
	Yes	39 (25.8%)	96 (63.6%)	16 (11.5%)				
Autoimmune disease	No	170 (24.6%)	441 (63.8%)	80 (11.6%)	1.73	1.88	1.09	0.67
	Yes	4 (36.4%)	6 (54.5%)	1 (9.1%)				
Age	18–50	166 (24.5%)	432 (63.7%)	80 (11.8%)	1.39	3.86	2.78	0.39
	>50	8 (33.3%)	15 (62.5%)	1 (4.2%)				
Second dose								
Sex	Male	70 (38.3%)	94 (51.4%)	19 (10.4%)	0.59	0.66	1.12	0.016
	Female	138 (27%)	316 (61.8%)	57 (11.2%)				
Allergies	No	178 (30.2%)	352 (59.7%)	60 (10.2%)	1.02	0.63	0.62	0.29
	Yes	30 (28.8%)	58 (55.8%)	16 (15.4%)				
Chronic disease	No	186 (29.9%)	375 (60.3%)	61 (9.8%)	1.27	0.48	0.38	0.01
	Yes	22 (30.6%)	35 (48.6%)	15 (20.8%)				
Medications	No	171 (31.4%)	324 (59.4%)	50 (9.2%)	0.82	0.42	0.51	0.01
	Yes	37 (24.8%)	86 (57.7%)	26 (17.4%)				
Autoimmune disease	No	207 (30.3%)	403 (59%)	73 (10.7%)	0.28	0.12	0.42	0.11
	Yes	1 (9.1%)	7 (63.6%)	3 (27.33%)				
Age	18–50	201 (30%)	398 (59.4%)	71 (10.6%)	1.16	0.49	0.43	0.28
	>50	7 (29.2%)	12 (50%)	5 (20.8%)				
Third dose								
Sex	Male	16 (40%)	21 (52.5%)	3 (7.5%)	0.48	1.50	3.14	0.11
	Female	24 (25.8%)	66 (70.9%)	3 (3.2%)				
Allergies	No	37 (33%)	69 (61.6%)	6 (5.4%)	0.31	-	-	0.05
	Yes	3 (14.3%)	18 (85.7%)	0 (0%)				
Chronic disease	No	34 (32.7%)	67 (64.4%)	3 (2.9%)	0.59	0.18	0.30	0.17
	Yes	6 (20.7%)	20 (68.9%)	3 (10.3)				
Medications	No	27 (33.3%)	52 (64.2%)	2 (2.5%)	0.72	0.24	0.34	0.27
	Yes	13 (25%)	35 (67.3%)	4 (7.7%)				
Autoimmune disease	No	39 (30.5%)	83 (64.8%)	6 (4.7%)	0.53	-	-	0.67
	Yes	1 (20%)	4 (80%)	0 (0%)				
Age	18–50	5 (27.8%)	12 (66.7%)	1 (5.6%)	1.12	1.40	1.25	0.96
	>50	35 (30.4%)	75 (65.2%)	5 (4.3%)				

**Table 3:** Parameters associated with the magnitude of the side effects of the ChAdOx1-S vaccine.

Parameters		Side Effect Magnitude; n (%)			Odds Ratio			*p* Value
		None	Mild	Strong	None/ Mild	None/ Strong	Mild/ Strong	
First dose								
Sex	Male	9 (30.0%)	19 (63.3%)	2 (6.7%)	0.51	0.25	0.48	0.24
	Female	10 (16.7%)	41 (68.3%)	9 (15%)				
Allergies	No	17 (23%)	50 (67.6%)	7 (9.5%)	0.59	0.21	0.35	0.19
	Yes	2 (12.5%)	10 (62.5%)	4 (25%)				
Chronic disease	No	15 (22.1%)	44 (64.7%)	9 (13.2%)	0.73	1.20	1.64	0.77
	Yes	4 (18.2%)	16 (72.7%)	2 (9.1%)				
Medications	No	13 (22%)	40 (67.8%)	6 (10.2%)	0.92	0.55	0.60	0.71
	Yes	6 (19.4%)	20 (64.5%)	5 (16.1%)				
Autoimmune disease	No	19 (21.3%)	59 (66.3%)	11 (12.4%)	0.00	-	-	0.78
	Yes	0	1 (100%)	0				
Age	18–50	16 (21.1%)	51 (67.1%)	9 (11.8%)	1.06	0.84	0.79	0.96
	>50	3 (21.4%)	9 (64.3%)	2 (14.3%)				
Second dose								
Sex	Male	13 (43.3%)	17 (56.7%)	0	0.30	-	-	0.01
	Female	10 (16.9%)	43 (72.9%)	6 (10.2%)				
Allergies	No	19 (26%)	53 (72.6%)	1 (1.4%)	1.59	0.04	0.03	0.000075
	Yes	4 (25%)	7 (43.8%)	5 (31.3%)				
Chronic disease	No	16 (23.9%)	46 (68.7%)	5 (7.5%)	1.44	2.19	1.52	0.71
	Yes	7 (31.8%)	14 (63.6%)	1 (4.5%)				
Medications	No	15 (25.9%)	40 (69%)	3 (5.2%)	1.07	0.53	0.50	0.72
	Yes	8 (25.8%)	20 (64.5%)	3 (9.7%)				
Autoimmune disease	No	23 (26.1%)	59 (67%)	6 (6.8%)	-	-	-	0.78
	Yes	0	1 (100%)	0				
Age	18–50	17 (22.7%)	53 (70.7%)	5 (6.7%)	2.67	1.76	0.66	0.27
	>50	6 (42.9%)	7 (50%)	1 (7.1%)				

## Data Availability

The results obtained in the study are included in the figures, tables and Supplementary Materials. Any inquiries can be addressed to the corresponding author.

## Supplementary Materials

The following supporting information can be downloaded at: https://www.mdpi.com/article/10.3390/vaccines12010104/s1. Table S1: Characteristics of the participants; Table S2: Type and number of doses of the anti-SARS-CoV-2 vaccines administrated to the participants; Table S3: The association between having a history of allergy and developing allergy upon vaccination; Table S4: The infection with the SARS-CoV-2 and the magnitude of the side effects after the anti-SARS-CoV-2 vaccination; Table S5: The infection with the SARS-CoV-2 and the magnitude of the side effects after the BNT162b2 vaccine; Table S6: The infection with the SARS-CoV-2 and the magnitude of the side effects after the ChAdOx1-S vaccine. Supplementary Materials online: https://zenodo.org/records/10450867 (accessed on 2 January 2024).
