# Supplementary material for: Association of the Magnitude of Anti-SARS-CoV-2 Vaccine Side Effects with Sex, Allergy History, Chronic Diseases, Medication Intake, and SARS-CoV-2 Infection
Source: Vaccines (Basel). 2024 Jan 20;12(1):104. doi: 10.3390/vaccines12010104 (PMC10820381; doi:10.3390/vaccines12010104)
Supplement: Supplementary file 1 [file vaccines-12-00104-s001.zip › vaccines-2830908-supplementary.pdf]

Parameters		N (%)
**Sex**	**Females**	616 (73.25%)
	**Males**	225 (26.75%)
**Age**	**18-29 years**	431 (51.37%)
	**30-39 years**	218 (25.92%)
	**40-49 years**	146 (17.24%)
	**≥50 years**	46 (5.47%)
**Chronic diseases**	**Total**	136 (11.89%)
	**Type 1 diabetes mellitus**	6 (0.71%)
	**Type 2 diabetes mellitus**	13 (1.55%)
	**Hypertension**	23 (2.7%)
	**Type 1 diabetes mellitus and hypertension**	1 (0.12%)
	**Type 2 diabetes mellitus and hypertension**	2 (0.24%)
	**Autoimmune diseases**	12 (1.43%)
	**Other**	52 (6.2 %)

Supplementary Table S1. Characteristics of the participants.

Vaccine	First dose N (%)	Second dose N (%)	Third dose N (%)
**BNT162b2**	702 (83.5%)	673 (81.2%)	101 (68.2%)
**ChAdOx1-S**	92 (66.2%)	98 (11.8%)	11 (7.4%)
**Sinopharm**	2 (0.24%)	3 (0.36%)	0
**MRNA-1273**	0	1 (0.12)	2 (1.35%)
**Ad26.COV2. S**	1 (0.12%)	0	0
**Sputnik V**	1 (0.12%)	1 (0.12%)	0
**Sinovac-Coronavac**	1 (0.12%)	0	0
**Other**	1 (0.12%)	2 (0.24%)	1 (0.7%)
**Do not know**	41 (4.9%)	51 (6.2%)	33 (22.3%)

Supplementary Table S2. Type and number of doses of the anti-SARS-COV-2 vaccines administrated to the participants

First Dose All Vaccines; N (%)				
	**Did not Develop Allergy**	**Developped Allergy**	**Odds Ratio**	***P*-Value**
**No Allergy**	709 (99.44%)	4 (0.56%)	0.11	0.000062
**Having Allergy**	120 (95.24%)	6 (4.76%)		
**Second Dose All Vaccines; N (%)**				
	**Did not Develop Allergy**	**Developped Allergy**		
**No Allergy**	698 (99.43%)	4 (0.57%)	0.23	0.041
**Having Allergy**	123 (97.62%)	3 (2.38%)		
**First Dose BNT162b2; N (%)**				
	**Did not Develop Allergy**	**Developped Allergy**		
**No Allergy**	595 (99.5%)	3 (0.5%)	0.1	0.000134
**Having Allergy**	99 (95.19%)	5 (4.81%)		
**Second Dose BNT162b2; N (%)**				
	**Did not Develop Allergy**	**Developped Allergy**		
**No Allergy**	587 (99.49%)	3 (0.51%)	0.17	0.016
**Having Allergy**	101 (97.12%)	3 (2.88%)		
**First Dose ChAdOx1-S; N (%)**				
	**Did not Develop Allergy**	**Developped Allergy**		
**No Allergy**	73 (98.65%)	1 (1.35%)	0.21	0.23
**Having Allergy**	15 (93.75%)	1 (6.25%)		
**Second Dose ChAdOx1-S; N (%)**				
	**Did not Develop Allergy**	**Developped Allergy**		
**No Allergy**	73 (100%)	0 (0%)	0	0.23
**Having Allergy**	15 (93.75%)	1 (6.25%)		

Supplementary Table S3. The association between having a history of allergy and developing allergy upon vaccination

Number of infections with SARS-CoV-2	Side effect magnitude; N (%)			Odds Ratio			*P* value
	None	Mild	Strong	None/ Mild	None/ Strong	Mild/ Strong	
**First dose**							
None	99 (24.5%)	259 (64.1%)	46 (11.4%)	0.99	0.94	0.95	0.965
One	105 (24.1%)	278 (63.9%)	52 (12%)				
None and one	204 (24.3%)	537 (64%)	98 (11.7%)	1.24	1.44	1.16	0.41
Two	33 (28.9%)	70 (61.4%)	11 (9.7%)				
None	99 (24.5%)	259 (64.1%)	46 (11.4%)	1.23	1.39	1.13	0.31
Two	33 (28.9%)	70 (61.4%)	11 (9.7%)				
None	99 (24.5%)	259 (64.1%)	46 (11.4%)	1.04	1.02	0.98	0.97
One and Two	138 (25.1%)	348 (63.4%)	63 (11.5%)				
One	105 (24.1%)	278 (63.9%)	52 (12%)	1.25	1.49	1.19	0.31
Two	33 (28.9%)	70 (61.4%)	11 (9.7%)				
**Second dose**							
None	143 (35.6%)	226 (56.4%)	32 (8%)	0.62	0.44	0.70	0.001
One	105 (24.6%)	268 (62.8%)	54 (12.6%)				
None and one	248 (29.9%)	494 (59.7%)	86 (10.4%)	0.58	0.36	0.63	0.002
Two	21 (18.6%)	72 (63.7%)	20 (17.7%)				
None	143 (35.6%)	226 (56.4%)	32 (8%)	0.46	0.23	0.51	0.000189
Two	21 (18.6%)	72 (63.7%)	20 (17.7%)				
None	143 (35.6%)	226 (56.4%)	32 (8%)	0.59	0.38	0.65	0.001
One and Two	126 (23.3%)	340 (63%)	74 (13.7%)				
One	105 (24.5%)	268 (62.9%)	54 (12.6%)	0.74	0.54	0.73	0.068
Two	21 (18.6%)	72 (63.7%)	20 (17.7%)				
**Third dose**							
None	21 (32.8%)	42 (65.6%)	1 (1.56%)	0.97	0.29	0.29	0.54
One	18 (31%)	37 (63.8%)	3 (5.2%)				
None and one	39 (32%)	79 (64.8%)	4 (3.2%)	0.89	0.18	0.20	0.054
Two	7 (25.9%)	16 (59.3%)	4 (14.8%)				
None	21 (32.8%)	42 (65.6%)	1 (1.6%)	0.88	0.08	0.10	0 .039
Two	7 (25.9%)	16 (59.3%)	4 (14.8%)				
None	21 (32.8%)	42 (65.6%)	1 (1.56%)	0.94	0.17	0.18	0.199
One and Two	25 (29.4%)	53 (62.4%)	7 (8.2%)				
One	18 (31%)	37 (63.8%)	3 (5.2%)	0.90	0.29	0.32	0.32
Two	7 (25.9%)	16 (59.3%)	4 (14.8%)				

Supplementary Table S4. The infection with the SARS-CoV-2 and the magnitude of the side effects after the anti-SARS-CoV-2 vaccination.

Number of infections with SARS-CoV-2	Side effect magnitude; N (%)			Odds Ratio			*P* value
	None	Mild	Strong	None/ Mild	None/ Strong	Mild/ Strong	
**First dose**							
**None**	85 (25.2%)	211 (62.6%)	41 (12.2%)	0.81	0.88	1.08	0.85
**One**	60 (21.7%)	184 (66.4%)	33 (11.9%)				
**None and one**	145 (23.6%)	395 (64.3%)	74 (12.1%)	1.52	2.11	1.39	0.125
**Two**	29 (33%)	52 (59%)	7 (8%)				
**None**	85 (25.2%)	211 (62.6%)	41 (12.2%)	1.38	2.00	1.44	0.248
**Two**	29 (33%)	52 (59%)	7 (8%)				
**None**	85 (25.2%)	211 (62.6%)	41 (12.2%)	0.94	1.07	1.15	0.824
**One and Two**	89 (24.4%)	236 (64.6%)	40 (11%)				
**One**	60 (21.7%)	184 (66.4%)	33 (11.9%)	1.71	2.28	1.33	0.082
**Two**	29 (33%)	52 (59%)	7 (8%)				
**Second dose**							
**None**	119 (35.4%)	188 (56%)	29 (8.6%)	0.63	0.46	0.73	0.005
**One**	89 (24.9%)	222 (62%)	47 (13.1%)				
**None and one**	208 (30%)	410 (59.1%)	76 (10.9%)	0.73	0.41	0.56	0.012
**Two**	19 (21.8%)	51 (68.6%)	17 (19.6%)				
**None**	119 (35.4%)	188 (56%)	29 (8.6%)	0.59	0.27	0.46	0.003
**Two**	19 (21.8%)	51 (68.6%)	17 (19.6%)				
**None**	119 (35.4%)	188 (56%)	29 (8.6%)	0.62	0.41	0.66	0.004
**One and Two**	108 (24.3%)	273 (61.3%)	64 (14.4%)				
**One**	89 (24.9%)	222 (62%)	47 (13.1%)	0.93	0.59	0.64	0.118
**Two**	19 (21.8%)	51 (68.6%)	17 (19.6%)				
**Third dose**							
**None**	18 (31.6)	38 (66.7%)	1 (1.8%)	0.97	0.30	0.31	0.56
**One**	16 (29.6%)	35 (64.8%)	3 (5.6%)				
**None and one**	34 (30.6%)	73 (65.8%)	4 (3.6%)	0.92	0.35	0.38	0.52
**Two**	6 (27.3%)	14 (63.6%)	2 (9.1%)				
**None**	18 (31.6%)	38 (66.7%)	1 (1.8%)	0.90	0.17	0.18	0.306
**Two**	6 (27.3%)	14 (63.6%)	2 (9.1%)				
**None**	18 (31.6%)	38 (66.7%)	1 (1.8%)	0.95	0.24	0.26	0.411
**One and Two**	22 (28.9%)	49 (64.5%)	5 (6.6%)				
**One**	16 (29.6%)	35 (64.8%)	3 (5.6%)	0.94	0.56	0.60	0.848
**Two**	6 (27.3%)	14 (63.6%)	2 (9.1%)				

Supplementary Table S5. The infection with the SARS-CoV-2 and the magnitude of the side effects after the BNT162b2 vaccine.

Number of infections with SARS-CoV-2	Side effect magnitude; N (%)			Odds Ratio			*P* value
	None	Mild	Strong	None/ Mild	None/ Strong	Mild/ Strong	
**First dose**							
None	9 (23.1%)	27 (69.2%)	3 (7.7%)	1.29	0.75	0.58	0.509
One	9 (26.5%)	21 (61.8%)	4 (11.8%)				
None and one	18 (24.3%)	48 (64.9%)	7 (10.8%)	0.22	0.13	0.58	0.233
Two	1 (12.4%)	12 (81.3%)	3 (6.3%)				
None	9 (23.1%)	27 (69.2%)	3 (7.7%)	0.25	0.11	0.44	0.213
Two	1 (12.4%)	12 (81.3%)	3 (6.3%)				
None	9 (23.1%)	27 (69.2%)	3 (7.7%)	0.91	0.48	0.52	0.509
One and Two	10 (19.6%)	33 (64.7%)	7 (15.7%)				
One	9 (26.5%)	21 (61.8%)	4 (11.8%)	0.19	0.15	0.76	0.267
Two	1 (12.4%)	12 (81.3%)	3 (6.3%)				
**Second dose**							
None	12 (30.8%)	26 (66.7%)	1 (2.6%)	0.93	0.19	0.20	0.293
One	9 (26.5%)	21 (61.8%)	4 (11.8%)				
None and one	21 (28.8%)	47 (64.4%)	5 (6.8%)	0.34	0.48	1.38	0.386
Two	2 (12.4%)	13 (81.3%)	1 (6.3%)				
None	12 (30.8%)	26 (66.7%)	1 (2.6%)	0.33	0.17	0.50	0.324
Two	2 (12.4%)	13 (81.3%)	1 (6.3%)				
None	12 (30.8%)	26 (66.7%)	1 (2.6%)	0.70	0.18	0.26	0.293
One and Two	11 (22%)	34 (68%)	5 (10%)				
One	9 (26.5%)	21 (61.8%)	4 (11.8%)	0.36	0.89	2.48	0.386
Two	2 (12.4%)	13 (81.3%)	1 (6.3%)				

Supplementary Table S6. The infection with the SARS-CoV-2 and the magnitude of the side effects after the ChAdOx1-S vaccine.
